# Monthly Summary

**Published:** 1875-01

**Authors:** 


### MONTHLY SUMMARY.

*On Intra-buccal Resection of the Inferior Dental Nerve after Paravicini's Method; Recovery.*—Dr. Mosetig-Moorhof, of Vienna, says (*Wiener Medizinische Wochenscrift*, March 21, 1874) that the various methods for the division of the inferior dental nerve, which have been proposed in cases of severe neuralgia, are as follows. The vast superiority of the process invented by Paravicini will be at once perceived.

1. Division of the cheek through its entire thickness, at a spot corresponding with the anterior edge of the ramus of the jaw, without dividing the mucous membrane.

2. Division of the cheek at a spot corresponding with the sigmoid notch, in a direction upwards.

3 Division of the soft parts over the posterior border of the ramus of the jaw, in a direction from behind and below, inwards and upwards.

4. Removal of a portion of the angle of the jaw.

5. Trephining the ramus above the commencement of the canal after division of the soft parts.

The method adopted in the case reported by Dr. Mosetig-Moorhof was as follows : The mouth being widely opened, the mucous membrane is divided along the anterior border of the ramus of the jaw, and the finger is directed between the bone and the internal pterygoid muscle to the lingula. The separation of the inferior maxillary from the gustatory nerve is not difficult, since the former passes into the canal and the latter does not. The operation, however, is so far difficult, since the eye cannot be of any assistance, and the operator must trust to his power of touch, and has to work in a very constricted and narrow space ; but the result is great relief to the patient, and no disfigurement at all as there is after the before-mentioned methods.

Dr. Mosetig-Moorhof operated on his patient on July 31, and having found the lingula, divided the internal lateral ligament of the jaw. The nerve and artery were secured by a thread, which was passed round them by means of a small aneuism-needle (it being absolutely necessary to fix them in order to cut through the nerve centrally) and so removed a piece of it. A piece of nerve about four inches long was removed. The reaction which followed a cessation of the neuralgia, and a want of feeling of the right side of the lower jaw, was trifling. Beyond a swelling of the side of the face and pharynx, with some slight difficulty of swallowing, the patient had little to complain of.

The patient was perfectly well by the end of January, and had lost all symptoms of neuralgia.—*London Med. Record.*

---

*Treatment of Dog Bites*—If measures are not adopted to stamp out rabies in the dog, then we must endeavor to find a means of treating the bite in such a way as to render it, if possible, harmless. When we consider this subject, difficulties assail us which seem well nigh insurmountable, and make us doubtful of ever attaining the desired end. When we reflect on the rapidity with which substances are absorbed, and travel the whole round of the circulation, we are inclined to question the efficacy of any treatment after the bite is made. It is, however, possible that the peculiar poison of rabies may have a slow rate of absorption, and so give us some chance of removing it from the wound, or rendering it by some means innocuous. Hippocrates of old, in his wisdom, which we all admire, said that the physician must "have two special objects in view with regard to diseases, namely, to do good or to do no harm." That is a maxim the ventilation of which might be of eminent service, even in this enlightened age, in general practice, and it is particularly appropriate when we come to consider what must be done with the bite of a mad dog.

Tetanus is a disease which, in its symptoms, has a peculiar resemblance to hydrophobia. Spasms occur in each on the slightest irritation of any of the organs of sense; both are peculiarly diseases of the nervous system; and in neither, on post-mortem examination, is there anything very definite to account for death. Tetanus we know to be caused by a wound, more especially by a wound which does not heal quickly. Hydrophobia is not supposed to be caused by the wound, but by the saliva, in which some virus exists. Nevertheless, it would seem right that we should not follow any treatment which can retard the healing process.

The principal indications, then, for the treatment of a bite are:—

1. As soon as possible to remove the virus from the wound or render it innocuous.
2. To get the part healed as speedily as possible.

With regard to the first indication, so soon as the bite is inflicted it is not likely that a surgeon can be at hand, and so it would be well for the patient to suck the parts vigorously, if they are within reach. That will encourage bleeding, and tend to remove any saliva that may be in the parts. A ligature between the wound and the heart might also be applied to encourage bleeding. When the surgeon is consulted I would advise, when the dog is known to be rabid, excision of the bitten parts where practicable ; afterwards, aud where excision cannot be performed, I would wash out or syringe the parts freely with a saturated solution of carbolic acid in very hot water, and get the wound healed as soon as possible.

By that means I think we may hope to remove by excision or destroy by the carbolic acid any virus in the wound, at the same time expedite the healing process. By such a method of treatment we can do no harm, and from the well known properties of carbolic acid, we may hope that it will do some good—*London Med. Times & Gazette.*

---

*Treatment of Malignant Pustule.*—The Paris correspondent of the *Irish Hospital Gazette* states that at a meeting, lately, of the Academy of Sciences, M. Bouley presented a memoir by M. Cezard, on what he terms the anti-virulent method of treating charbon, or malignant pustule, basing his theory on the experiments performed some short time ago by M. Davaine, illustrating the anti virulent properties of certain chemical agents. According to the author of this paper, iodine is considered the best antidote against the poison or virus of charbon, or malignant pustule He states that a dose of one-twelfth of a milligramme of iodine is sufficient to destroy the virulence of the fluid of malignant pustule, that is to say, when mixed in a test tube ; but that it will take much less to prevent or even destroy the virulence of this terrible affection when the drug is introduced into the organism. M. Cézard informs us that an animal can support, without any inconvenience, the introduction in the blood, at one and the same time, of a quantity of iodine amounting in weight to more than one-five-thousandth part of the entire mass of blood, that is to say, more than is sufficient to destroy instantaneously the virulence of malignant pustule, when the latter exists, and to prevent its development, when once the virus is introduced into the organism. Iodine whether administered by the digestive tube or by hypodermic injections, is absorbed in substance, and preserves its special properties even in the blood. M. Cézard advises that the drug be administered in the form of iodide of iodine, that is, in the proportion of one part of iodine to two of the iodide of potassium, which renders it more soluble in water and mitigates its irritating properties. This method of treatment, he continued, is very efficacious, not only against the true malignant pustule when it has attained the stage characterized by œdema, but also before it reaches that period, and even against the symptomatic fever of malignant pustule. M. Cézard also employs iodine locally in this affection, in the form of subcutaneous injection of a solution in the proportion of one-five-hundredth, and in the form of lotions, in the proportion of one-hundredth of the iodide of iodine. If there be a slough this should be previously excised, in order to facilitate the action of the drug.—*Ibid* —*Nashville Journal of Medicine and Surgery.*

---

*To Keep Away Flies.*—The sick and wounded are, in warm weather, often dreadfully annoyed by fles, The suggestion of a French veterinary surgeon deserves trial at such times. He says that a simple method of preventing flies from annoying horses consists in painting the inside of the ears, or any other part especially troubled, with a few drops of empyræumatic oil of juniper. It is said that the odor of this substance is unendurable to flies, and that they will keep at a distance from the parts so annointed. If this treatment should accomplish the alleged result, it will be a great blessing to introduce into the sick room.—*The Cincinnati Lancet and Observer.*

---

*Influence of Anœsthetics upon the Sexual Impressions of Females.*—A writer ("London Medical Clinic") says it is a well established fact, that occasionally, under the influence of ether or chloroform, an excitation of the sexual organs is produced, and a feeling is excited in the mind by this sensation which may make a woman believe that she has been subjected to violence. In illustration of this statement the writer says, that during delivery, he placed the woman under chloroform. The sexual sensations of the woman were so vivid that she accused him of having violated her. Yet her husband and a dozen women had been present the entire time of delivery. Other illustrations are given, from which the wise moral is deduced, "that physicians should never administer ether or chloroform except in presence of witnesses."—*American Medical Weekly.*

---

*Electricity a Substitute for Gas or Coal-Oil.*—The danger of kerosene may possibly be done away with by the invention of a Russian who claims to have discovered a process for producing light by electricity, which is thus described: A small tube of glass, not more than six inches in length, is filled with a pencil of charcoal; the air is exhausted and the tube hermetically sealed. A moderate current of electricity is then passed through the charcoal from an ordinary electro-magnetic machine, causing it to glow with a very brilliant, but at the same time a soft light. It is stated that the charcoal is not perceptibly consumed by the process, but will last for an indefinite period, and that the strength of the current required is so small that two hundred of these lights, at a considerable distance apart, can be easily maintained by a single machine. The inventor claims that he can light the whole city of St. Petersburg, both street-lamps, stores, and private residences, by a single fifteen-horse-power machine, with no greater cost than that of running the machine. Moreover, all the lamps in the city would be lighted at the same moment, and private lights would need no attention, except the shutting off of the current from the house when desired.—*Tenn. Phar. Gazette.*

---

*Ante-natal Development of Nine Teeth.*—At a recent meeting of the Philadelphia Obstetrical Society, Dr. C. H. Thomas related the case of an infant which, though normally constituted otherwise, exhibited nine perfect teeth when born. In addition to these, a number of small, whitish nodules could be seen and felt along the line of the gums, above and below, lying underneath the mucous membrane, and evidently marking the location of all the other deciduons teeth.—*Amer. Jour. of Obstetrics.*

---

*Celluloid.*—It is said that several companies have been formed in this city for the manufacture of different objects from celluloid, the new substitute for ivory. As originally prepared it consisted of a combination of soluble cotton and ether or alcohol, but it was subsequently ascertained that a still more satisfactory result could be obtained by the addition of camphor to the alcohol; and finally comphor alone was mixed with the ground cotton pulp, which hardens in drying and becomes "celluloid." Probably this substance will also find various uses in surgery,—*Phil. Med. and Surg.*

---

*Handy Method of Examining Nervous Tissues Microscopically.*—D. Tuke gives the following convenient method for examining nervous tissues. It is not intended to take the place of the old processes, but to supplement them:

A portion of nerve tissue, the size of a large pin's head, is taken from a thoroughly defined locality. It is gently flattened under a covering glass on a side. The covering glass is then removed, and a drop of "Judson's simple (aniline) magenta dye," diluted with eight drops of water, is applied to the mass. With a needle this dye is carefully mixed with the nerve matter. The preparation is covered with a clean glass, and gently pressed until light can pass through it. Specimens so prepared will clearly exhibit the nerve cells, nuclei of the neurologia, and the blood-vessels, tinted a deep crimson color, leaving the other tissues unaffected.—*British Med. Journal.*

